# Impact event and orofacial pain amid the COVID-19 pandemic in Brazil: a cross-sectional epidemiological study

**DOI:** 10.1590/1678-7757-2021-0122

**Published:** 2021-10-01

**Authors:** Fernanda Pereira DE CAXIAS, Flávia Regina Florencio de ATHAYDE, Marcella Santos JANUZZI, Larissa Viana PINHEIRO, Karina Helga Leal TURCIO

**Affiliations:** 1 Universidade Estadual Paulista Faculdade de Odontologia Departamento de Materiais Odontológicos e Prótese Araçatuba Brasil Universidade Estadual Paulista (UNESP), Faculdade de Odontologia, Departamento de Materiais Odontológicos e Prótese,Araçatuba, Brasil.; 2 Universidade Estadual Paulista Faculdade de Medicina Veterinária Departamento de Produção e Saúde Animal Araçatuba Brasil Universidade Estadual Paulista (UNESP), Faculdade de Medicina Veterinária, Departamento de Produção e Saúde Animal, Araçatuba, Brasil.

**Keywords:** Depression, Anxiety, Stress, Psychological, Coronavirus Infections, Pandemics, Facial Pain

## Abstract

**Objectives:**

This study aims to assess the impact of social isolation, due to the Covid-19 pandemic, on mental health, Temporomandibular Disorder (TMD) and orofacial pain in men and women.

**Methodology:**

Individuals living in Brazil answered an online questionnaire on their sociodemographic and behavioral aspects, emotional scale (DASS-21), Impact of Event Scale, and Pain Screener in Temporomandibular Disorders (TMD-Pain Screener) during June 2020. Descriptive statistical analyses and logistic and linear regressions were applied (5% significance).

**Results:**

Overall, 2301 individuals were included, 89.1% practiced social isolation, 72.6% were employed/studying, at least 15% presented severe or extremely severe levels of emotional distress and presence of powerful (34.1%) and severe impact event (15%). During the outbreak, 53.2% perceived feeling worse and 31.8% reported that orofacial pain started or worsened after the pandemic outbreak. Gender was associated with “social class” (P=0.036), “pain/stiffness in the jaw on awakening” (P=0.037), “change of pain during jaw habits” (P=0.034) and “perception of change in the situations mentioned in the TMD-Pain Screener” (P=0.020), “depression” (P=0.012), “anxiety” (P=0.006) and “impact of the event” (P=8.3E-11). Social isolation had a lesser chance to change the routine, to be practiced by the unemployed/not studying, and to be practiced by men (all with P<0.001). Associations were found between social class and all subscales of the DASS-21 and IES, all with P<0.001.

**Conclusions:**

The practice of social isolation has social determinants. High levels of psychological and event impacts were detected. The presence of orofacial pain seemed to increase during the health crisis, and there were gender differences in the response to the COVID-19 pandemic.

## Introduction

Severe acute respiratory syndrome coronavirus 2 (SARS-CoV-2), known as COVID-19, originated in China, caused a health crisis worldwide, being classified as a pandemic by the World Health Organization (WHO).^1^ Measures of social isolation and quarantine were applied worldwide^1^ aiming to decelerate the contagion. These changes in routine can cause helplessness and feelings of abandonment,^1^ which can negatively influence the mental health of individuals.

Johns Hopkins University (USA) registered 16,624,480 cases with 465,199 deaths, in Brazil, on June 2^nd^, 2021.^2^ Brazil became the epicenter, with 887,850 new cases and 30,270 deaths registered in June 2020, according to official data from the Brazilian Ministry of Health.^3^ The country is in development, counting on a health system incapable of offering enough hospital beds to meet the high demand, allowing fear and anxiety – further aggravated by misinformation – to change the overall behavior of the population.^4^

Regarding Dentistry, it is important to emphasize the multifactorial nature of temporomandibular disorders (TMDs), including psychosocial causes.^5^ The Diagnostic Criteria for TMD Questionnaire (DC/TMD) acknowledges the importance of the psychological and social aspects of pain.^5^ It is possible to speculate an increase of severe psychological and physiological disorders, of interest to Dentistry, during the pandemic. Specialists forecast that the pandemic’s influence on psychological factors may increase the risk of development, or worsening, of symptoms related to TMDs.^6^

Several studies relating the difference between gender and the presence of orofacial pain and TMDs have been conducted,^7^ reporting that women have a higher prevalence of presenting almost every painful symptom in the orofacial region. Some studies have shown that women report more painful experiences and have a more negative response to pain.^8^ Many aspects of these differences should be noted, such as behavior, perception, pain tolerance, and the structural differences of the masticatory system. Emotions and negative stress influence pain perception and expectation. Coping ability (i.e., perceived stress) and behavior choice (exposition, fear-escape, adaptation, and no adaptation) also influence the pain experience.^9^The strategy of coping and cognition seems to be different between men and women, according to El-Shormilisy, Strong, and Meredith^10^ (2015), who reported a maladaptive coping strategy and high catastrophizing levels in women. This can be important because women are twice as likely to develop TMD compared to men.^11^

This study aims to assess the influence of social isolation due to the Covid-19 pandemic on mental health and on TMD and orofacial pain in men and women. The hypotheses are that there could be sociodemographic determinants for the practice of social isolation and high levels of depression, anxiety, stress; and that there is a possible relation between the emotional influence of the COVID-19 pandemic and frequent presence of orofacial pain, with notable differences between genders.

## Methodology

### Study design

The study was performed using an epidemiological cross-sectional and analytic design. Participants answered an online questionnaire and were encouraged to pass it to others. The questionnaire was sent to different social groups through social media, emails, and message applications aiming to decrease the risk of homogeneity of participants and consequent bias. Answers were collected in June 2020, amid the peak of the first wave of the COVID-19 crisis in Brazil.

### Ethical considerations

This study was approved by the Ethics Committee of Human Research of the School of Dentistry in Araçatuba, UNESP (opinion number: 32483020.7.0000.5420). The participants received information about the study and ethical principles. They had access to the informed consent form and choose to participate in the research by selecting the appropriate option before starting the questionnaire (“I do not wish to participate” or “I wish to participate in a free and informed way”). The recommendations of the Ethics Committee were followed, and the principles of Helsinki^12^ were applied.

### Participants

Adults resident in Brazil were invited to participate in the study. The participants had to be 18 years of age or older, able to read and understand Brazilian Portuguese and to have the cognitive ability to comprehend and answer the questions. The participants could be living in any of the five regions of Brazil, belong to any social class, and have any education level, provided that they were literate. Individuals who did not complete the questionnaire, underaged, as well as duplicated forms were excluded from the study.

### Questionnaire

The questionnaire was developed and applied using Google Forms software (Google LCC, California, USA). It was composed of 53 questions that collected sociodemographic and general health information, emotional state, and pain amid the COVID-19 pandemic. The Brazilian Portuguese versions of the questionnaires Depression, Anxiety and Stress Scale (DASS-21),^13^ Impact of Event Scale (IES),^14^ and the Pain Screener from the Diagnostic Criteria for TMD (DC/TMD)^15^ were also applied.

Sociodemographic and general health questions collected information on the practice of social isolation, social class, age, gender, changes in routine, and if the participant was employed/studying.

The DASS-21 had 21 questions that assessed symptoms of depression and anxiety in an interactive and empirically oriented way, besides assessing stress.^13^ The participants had to select how much each item applied to them during the seven days before the interview and the answers had different levels of severity.^16^ This questionnaire allowed the classification of severity of problems into normal, mild, moderate, severe, and extremely severe.^13^ At the end of the DASS-21 questionnaire, a question was added to compare the perception of the situations mentioned in the questionnaire with those of the period before the pandemic.

The IES had 15 questions that assessed the stress subjected to life events, without focusing on one specific event.^14^This questionnaire assessed the frequency of each item to the participant, during the seven days before the interview. In this study, the heading was adapted to focus on the COVID-19 pandemic. The impact event could be: no meaningful impact; impact event—you may be affected; powerful impact event—you are certainly affected; severe impact event—this is capable of altering your functional ability.^17^

The TMD-Pain Screener is part of the DC/TMD questionnaire^15^ and assessed the presence of orofacial pain during the 30 days before the interview. It had three questions with different options of answer, related to the presence of pain in the jaw and temple regions in different everyday situations.^18^ At the end of the TMD-Pain Screener questionnaire, a question was added to compare the perception of the situations mentioned in the questionnaire with those of the period before the pandemic. Another question was added to collect information regarding pain lasting more than three months.

### Statistical Analysis

Sample size was estimated to include 1,412 participants, considering the estimated prevalence of chronic orofacial pain in 19% the population of this study, with α = 0.05, β = 0.2, and power = 0.8. This percentage was estimated based on a pilot study with 160 participants, in which this value was found in the question “Have you had pain in the region of the head and neck, mainly on the mandible and its joint for more than three months?”. The website https://clincalc.com/stats/samplesize.aspx was used to estimate sample size.

Descriptive analyses were performed for demographic data. The distribution and percentages were estimated. Quantitative statistical analyses were performed using R software (version 3.5.3; R Foundation for Statistical Computing, Austria). Logistic regression was performed with data regarding questions on the practice of social isolation and gender as independent variables. The remaining data were analyzed as dependent variables. The probability rates (odds ratio) with superior and inferior confidence limits (95% confidence interval) were estimated. Linear regression was applied for age, social class, and the DASS-21 and IES questionnaires, all with 5% significance.

## Results

### Qualitative data

In total, 2,352 responses were received in June 2020. From these, three were initially excluded because two individuals chose not to participate and one did not complete the questionnaire. Subsequently, the remaining responses were verified, 48 were identified as duplicates and, consequently, excluded. Thus, 2301 participants were included in the study, with a mean age of 41.4 years (range, 18–83 years). Among the 2,301 participants, 1,513 were females (65,8%), 537 males (23.3%) and 2 non-binary individuals (0.09%). Most participants declared to have practiced social isolation (n=2,052). On the other hand, 151 women (6.56%) and 98 men (4,25%) declared that they did not practice social isolation (n=249). Table 1 shows the distribution of demographic data according to practice of social isolation and gender.

**Table 1:** Distribution of demographic data according to practice of social isolation and gender

	Gender	Social Class		Presence of children		Daily routine*		Performance		Student		Worker	

With Social isolation	F	A	180	Yes	429	Home study/office	1218	Improved	248	Yes	348	Yes	1023
		B	475			No home study/office	177	Worsened	821				
		C	553										
		D	228	No	1084	Same routine	118	Same	444	No	1165	No	490
		E	77										
	M	A	78	Yes	166	Home study/office	414	Improved	95	Yes	83	Yes	435
		B	192			No home study/office	76	Worsened	254				
		C	171										
		D	77	No	371	Same routine	47	Same	188	No	454	No	102
		E	19										
	NB	A	1	Yes	1	Home study/office	2	Improved	2	Yes	0	Yes	2
		B	0			No home study/office	0	Worsened	0				
		C	0										
		D	0	No	1	Same routine	0	Same	0	No	2	No	0
		E	1										

Whithout social isolation	F	A	9	Yes	40	Home study/office	83	Improved	13	Yes	19	Yes	127
		B	52			No home study/office	37	Worsened	74				
		C	58										
		D	19	No	110	Same routine	30	Same	63	No	131	No	23
		E	12										
	M	A	15	Yes	44	Home study/office	43	Improved	9	Yes	12	Yes	85
		B	29			No home study/office	35	Worsened	35				
		C	41										
		D	11	No	55	Same routine	21	Same	55	No	87	No	14
		E	3										
	NB	A	0	Yes	0	Home study/office	0	Improved	0	Yes	0	Yes	0
		B	0			No home study/office	0	Worsened	0				
		C	0										
		D	0	No	0	Same routine	0	Same	0	No	0	No	0
		E	0										

F: Female; M: Male; NB: Non-Binary *The question was: "Have you changed your routine and started working and/or studying from home during the pandemic?"

Regarding the results for DASS-21 questionnaire, more than half of the participants were classified as “normal” in the three subscales (Depression n=1,239; Anxiety n=1,413 and Stress n=1,294). Results of “severe” and “extremely severe” counted at least 15% of participants for each subscale (severe: Depression n=162; Anxiety n=146 and Stress n=234; extremely severe: Depression n=186; Anxiety n=251 and Stress n=136).

The answers to the question “Do you feel that the situations mentioned above (DASS-21 questions) were different in the last week compared to with those of the period before the pandemic?” were: “Yes, I feel they have been worse” (n=1,224=53.2%), “Yes, I feel they have been better” (n=359=15.6%); “No, I have felt the same as I did before the pandemic” (n=718=31.2%).

Regarding the IES questionnaire, the distribution of scores was: no meaningful impact (n=443=19.2%), impact event—you may be affected (n=731=31.7%), powerful impact event—you are certainly affected (n=786=34.1%), and severe impact event—this is capable of altering your ability to function (n=359=15%).

Table 2 shows the distribution of answers for the TMD-Pain Screener. Answers to the question “If you answered yes to any of the questions above (TMD-Pain Screener), was this pain different from that of the period before the pandemic?” were: 1) “Yes, because the pain started after the pandemic” (n=284, 12.4%); 2) “Yes, the pain was present before the pandemic, but it has been worse” (n=445, 19.4%); 3) “Yes, the pain was present before the pandemic, but it has been better” (n=54, 2.3%); 4) “No, the pain is the same or I have not felt pain during the pandemic” (n=1518, 65.9%). Answers to the question “Have you had pain in the area of the head and neck, mainly on the mandible and its joint for more than three months?” were: “Yes” (n =679, 29.5%) and “no” (n=1622, 70.5%).

**Table 2:** Distribution for each question of TMD-Pain Screener questionnaire

1) In the last 30 days, how long did any pain last in your jaw or temple area on either side?		
Answer	Frequency	Percentage %
No pain	1418	61.6
Pain comes and goes	732	31.8
Pain is always present	151	6.6
2) In the last 30 days, have you had pain or stiffness in your jaw on awakening?		
Answer	Frequency	Percentage %
No	1518	70
Yes	783	30
3) In the last 30 days, did the following activities change any pain (that is, make it better or make it worse) in your jaw or temple area on either side?		
3.a Chewing hard or tough food		
Answer	Frequency	Percentage %
No	1846	80.3
Yes	455	19.7
3.b Opening your mouth or moving your jaw forward or to the side		
Answer	Frequency	Percentage %
No	1,776	77.2
Yes	525	22.8
3.c Jaw habits such as holding teeth together, clenching, grinding, or chewing gum		
Answer	Frequency	Percentage %
No	1,398	60.7
Yes	903	39.3
3.d Other jaw activities such as talking, kissing, or yawning		
Answer	Frequency	Percentage %
No	1,854	80.57
Yes	447	19.4

The questions and replies were copied and pasted from the English version of DC/TMD Assessment Instruments.^18^

### Quantitative data

The number of participants that declared to be non-binary for the question of gender was inexpressive (n=2); however, they were excluded from the quantitative analysis.

### Logistic regression

Associations were found between the “practice of social isolation” and “gender”, “changes in routine” and “employed/studying”, all with p<0.001. Men presented a 39% lesser chance of practicing social isolation. Individuals that practiced social isolation presented a 54% higher chance of having changes in routine, and individuals that were employed/studying had a 54% chance of not practicing social isolation (Table 3).

**Table 3:** Associations between practice of social isolation and gender, changes in routine, employment/studying

		No Practice of social isolation		Practice of social isolation		P-value	OR	CI 95%
		0		-1				
		Freq	%	Freq	%			

Gender	F	150	60.24	1,513	73.73	0.000	0.61	0.46 – 0.81
	M	99	39.76	537	26.17			
	Total	249		2,05				
Employed/studying	Yes	212	85.14	1,458	71.12	5.35E-05	0.46	0.31 - 0.66
	No	37	14.86	592	28.88			
	Total	249		2,05				
Changes in routine*	Yes	126	50.6	1,632	79.61	< 2e-16	0.46	0.39 – 0.55
	No	72	28.92	253	12.34			
	Routine did not change	51	20.48	165	8.05			
	Total	249		2,05				

Values found by logistic regression analysis. F: Female; M: Male; OR: Odds Ration; CI: Confience interval.

*The question was: "Have you changed your routine and started working and/or studying from home during the pandemic?"For this reason there are two negative answers, since they englobe the individuals who are not working or studying from home.

We found associations between “gender” and “social class” (p=0.036), “pain/stiffness in the jaw on awakening” (question 2 from the TMD- Pain Screener) (p=0.037), “change of pain during jaw habits” (question 3.c from the TMD-Pain Screener) (p=0.034) and “perception of change in the situations mentioned in the TMD-Pain Screener (p=0.020). (Table 4).

**Table 4:** : Associations between gender and sociodemographic, pain, DASS-21 and IES questionnaires data.

		Female (0)		Male (1)		p - value	OR	CI 95%
		Freq	%	Freq	%			

Social class	Class A	189	11.37	93	14.62	0.036	0.89	0.79 -0.99
	Class B	527	31.69	221	34.75			
	Class C	611	36.74	212	33.33			
	Class D	247	14.85	88	13.84			
	Class E	89	5.35	22	3.46			
Presence of pain in jaw and temporalis	No pain	929	55.86	488	76.73	0.070	0.79	0.61 - 1.01
	Pain comes and goes	597	35.9	134	21.07			
	Pain is always present	137	8.24	14	2.2			
Pain or stiffness in your jaw on awakening	No	916	55.08	156	24.53	0.037	1.36	1.02-1.81
	Yes	747	44.92	480	75.47			
3. a. chewing hard or tough food	No	1281	77.03	512	80.5	0.735	1.06	0.75 - 1.50
	Yes	382	22.97	124	19.5			
3.b opening or moving the jaw	No	1,226	73.72	548	86.16	0.708	0.93	0.65 - 1.33
	Yes	437	26.28	88	13.84			
3.c jaw habits	No	1,226	73.72	480	75.47	0.034	1.34	1.02 -1.76
	Yes	437	26.28	156	24.53			
3.d other jaw activities	No	1,304	78.41	548	86.16	0.101	0.76	0.55 - 1.05
	Yes	359	21.59	88	13.84			
Perception of change on situations (TMD-Pain Screener)	Yes, the pain was present before the pandemic, but it had been worse	397	23.87	48	7.55	0.020	1.23	1.03 - 1.47
	Yes, because the pain started after the pandemic	212	12.75	72	11.32			
	No, the pain is the same or I have not felt pain during the pandemic	1,016	61.09	500	78.62			
	Yes, the pain was present before pandemic, but it has been better	38	2.29	16	2.52			
Presence of pain for 3 months	No	1,102	66.27	519	81.6	0.102	0.79	0.59-1.04
	Yes	561	33.73	117	18.4			
Depression (DASS-21)	Normal	821	49.37	417	65.57	0.012	1.24	1.04 - 1.48
	Mild	252	15.15	71	11.16			
	Moderate	310	18.64	80	12.58			
	Severe	129	7.76	33	5.19			
	Extremely Severe	151	9.08	35	5.5			
Anxiety (DASS-21)	Normal	821	49.37	417	65.57	0.006	1.22	1.05 -1.41
	Mild	252	15.15	71	11.16			
	Moderate	310	18.64	80	12.58			
	Severe	129	7.76	33	5.19			
	Extremely Severe	151	9.08	35	5.5			
Stress	Normal	861	51.77	431	67.77	0.773	1.02	0.86 -1.22
(DASS-21)	Mild	251	15.09	85	13.36			
	Moderate	236	14.19	65	10.22			
	Severe	195	11.73	39	6.13			
	Extremely Severe	120	7.22	16	2.52			
Perception of situations mentioned on DASS 21	Yes, I feel they have been worse	263	15.81	96	15.09	0.938	0.99	0.84 -1.16
	Yes, I feel they have been better	956	57.49	268	42.14			
	No, I have felt the same as I did before the pandemic	444	26.7	272	42.77			
IES	No Meaningful Impact	240	14.43	202	31.76	8.3E-11	0.64	0.57 -0.73
	Impact Event	501	30.13	229	36.01			
	Powerful Impact Event	611	36.74	157	24.69			
	Severe Impact Event	311	18.70	48	7.55			

Values found by linear regression analysis.

We could not find associations between “gender” and “presence of pain in the jaw and temporalis” (p=0.070), “chewing hard food” (question 3.a from the TMD-Pain Screener) (p=0.735), “opening or moving the jaw” (question 3.b from the TMD-Pain Screener) (p=0.708), “other jaw activities” (question 3.d from the TMD-Pain Screener) (p=0.101), nor “presence of pain for three months” (p=0.102). Men presented a 28% lesser chance of having pain/stiffness in the jaw on awakening, were 1.34 times more likely to have changes of pain during jaw habits, and were 1.23 times more likely to perceive changes of situations mentioned in the TMD-Pain Screener (Table 4).

We found associations between “gender” and subscales of the DASS-21 questionnaire, “Depression” (p=0.012), “Anxiety” (p= 0.006) and “IES” (p=8.3E-11). No associations were found between the variable “gender” and subscale “Stress” (p=0.773) nor “perception of situations mentioned in the DASS-21” (p=0.938). The chances of an increase in severity of depression were 1.24 times higher in males than in females. Similarly, males showed a 1.22 times higher chance of increasing in the severity of anxiety. Each increase in severity of “IES” decreased by 36% the chance of it occurring in males (Table 4).

### Linear Regression

Associations were found between social class and all subscales of the DASS-21 and IES, all with p<0.001 (Table 5).

**Table 5:** Associations between social class, DASS-21 subscales and impact of the event (IES)

		Class A		Class B		Class C		Class D		Class E		F-statistic	P-value	Adjusted R2
	Level	Freq	%	Freq	%	Freq	%	Freq	%	Freq	%			

Depression (DASS-21)	Normal	195	69.15	444	59.36	417	50.67	139	41.49	43	38.74	88.59	< 2.2e-16	0.03672
	Mild	36	12.77	106	14.17	108	13.12	55	16.42	18	16.22			
	Moderate	36	12.77	111	14.84	158	19.2	66	19.7	19	17.12			
	Severe	5	1.77	44	5.88	67	8.14	31	9.25	15	13.51			
	Extremely Severe	10	3.55	43	5.75	73	8.87	44	13.13	16	14.41			
Anxiety (DASS-21)	Normal	215	76.24	500	66.84	466	56.62	170	50.75	60	54.05	61.58	6.45E-15	0.02569
	Mild	14	4.96	42	5.61	71	8.63	21	6.27	11	9.91			
	Moderate	28	9.93	112	14.97	122	14.82	53	15.82	17	15.32			
	Severe	12	4.26	37	4.95	53	6.44	37	11.04	7	6.31			
	Extremely Severe	13	4.61	57	7.62	111	13.49	54	16.12	16	14.41			
Stress (DASS-21)	Normal	192	68.09	459	61.36	435	435	156	46.57	50	45.05	53.53	3.50E-13	0.02235
	Mild	35	12.41	102	13.64	124	15.07	56	16.72	19	17.12			
	Moderate	35	12.41	96	12.83	109	13.24	43	12.84	18	16.22			
	Severe	12	4.26	55	7.35	101	12.27	53	15.82	13	11.71			
	Extremely Severe	8	2.84	36	4.81	54	6.56	27	8.06	11	9.91			
Impact of event	No Meaningful Impact	75	26.6	158	21.12	141	17.13	51	15.22	17	15.32	53.53	1.23E-06	0.02235
	Impact Event	102	36.17	232	31.02	256	31.11	104	31.04	36	32.43			
	Powerful Impact Event	74	26.24	244	32.62	300	36.45	117	34.93	33	29.73			
	Severe Impact Event	31	10.99	114	15.24	126	15.31	63	18.81	25	22.52			

Values found by linear regression analysis.

## Discussion

The hypotheses were confirmed, considering that we found sociodemographic determinants for the practice of social isolation, high levels of depression, anxiety, stress, and significant emotional influence of the COVID-19 pandemic; and associations between gender and sociodemographic factors, the pandemic and factors of TMD.

The world has faced several pandemics throughout history such as the Black Death, Tuberculosis, Spanish Influenza, and HIV/AIDS.^4^ However, the COVID-19 pandemic has been, so far, one of the worst, especially in Brazil. Data collection on behavior, psychological aspects, and TMD-related pain has become urgent due to the seriousness of the health crisis experienced worldwide.

A search on PubMed using the term “COVID-19” on May 4th, 2021 resulted in 130,554 articles, showing the significant impact of the pandemic in science. However, we could not find any study that assessed the correlation between impact of event and orofacial pain in the Brazilian population. A study by Wang, et al.^19^ (2020) analyzed, among other data, the psychological influence of the pandemic in China, which led to questions on the subject regarding the Brazilian population. The authors, however, did not collect data specific to Dentistry.^19^ These facts show the absence of studies in the specialty of temporomandibular disorders and orofacial pain and reinforce the necessity of research in the field of Dentistry during this historical period. Furthermore, one study showed the importance of socioeconomic determinants for the Covid-19 crisis and concluded that national and local contexts—such as gender, socioeconomic status, poverty, among others—are key factor to the outcome of Covid-19 crisis and to guide public health management.^20^ Our study brings novel information about the crisis in Brazil to help build strategies to attend to public demand.

The COVID-19 pandemic called for urgent changes in routine and behavior, especially regarding social relationships—a fundamental part of human life^21,22^that can affect health in various aspects, such as mental, behavioral, physical, and mortality risk.^22^ The absence of such relationships may be experienced by all sporadically; however, with the COVID-19 outbreak, isolation became necessary and mandatory, affecting peoples’ health and well-being.^23,24^ Our study assessed the practice of social isolation and found that 89.19% of participants declared to adhere to it. Nevertheless, we emphasize that the assessment was made thought the report of “yes” or “no”, presenting a possible limitation to this study, since each person considered isolation according to their own perception.

The WHO acknowledges the need for actions in mental health during the pandemic period, since an increase in symptoms of depression and anxiety have been reported in several countries.^25^ The study by Wang, et al.^19^ (2020) showed that, during the initial phase of the COVID-19 pandemic, more than half of the participants reported moderate to severe psychological impact and one-third reported moderate to severe anxiety. We should not only consider the psychological influence, but also pay close attention to the physiological effects that these psychiatric diseases can cause.

The prevalence of TMDs and the need for treatment differ among studies. The National Institute of Dental and Craniofacial Research affirmed—based on studies conducted in Europe, USA, and Hong Kong—that TMDs affect 5 to 15% of the population, with an approximate annual cost of 4 billion dollars.^26^ However, a meta-analysis affirmed that 16.2% of the population needs treatment and that this necessity varies according to the location of the study.^27^ It is important to emphasize that TMDs are not always accompanied by pain, but when they are painful, they can affect daily activities, psychosocial functions, and quality of life.^15^ The International Association for the Study of Pain affirms that headache and orofacial pain must last for at least three months to be considered chronic.^28^ Our study collected data regarding pain for at least three months (from the beginning of the COVID-19 crisis in Brazil to the moment of data collection) and found its presence in 29.5% of participants. Although most of participants did not feel TMD-related pain, this percentage is higher than normally found in other studies, demonstrating a possible increase in orofacial pain during the COVID-19 pandemic Moreover, regarding the situations mentioned on the TMD-Pain Screener, 12.4% of participants reported that their pain started after the pandemic and 19.4% that they had had pain before, but it became worse after the pandemic. This information is important for dental professionals that must be prepared to attend to the increasing treatment demand and to understand the psychological effect of the pandemic on psychosomatic pain. The increase in TMD-related chronic pain may also occur due to the social isolation and the paucity of dental treatments for several months.

In our study, 49.1% of individuals reported to have experienced the effect of the pandemic (powerful [34.1%] and severe impact events [15%]). At least 15% of individuals presented severe or extremely severe levels for each subscale of the DASS-21. These data corroborate the study by Wag, et al. (2020) that showed high rates of emotional alterations in the Chinese population during the pandemic.^19^Other authors also concluded that the COVID-19 outbreak has led to psychological problems such as stress, anxiety, depression, difficulty sleeping, and negative behaviors such as denial, anger, and fear, and that they deserve attention from public policies, as these changes may weaken strategies to control the COVID-19 crisis and lead to more mental health morbidities at a global level.^29^

Mowbray^30^ (2020) stated that the prevalence of post-traumatic stress disorder in the general population ranges from 4% to 41%. The same authors affirmed that the prevalence of major depression increased by 7% after the outbreak.^29^ They cited some factors that may increase the risk of developing these conditions: female gender, lower socioeconomic status, interpersonal conflicts, frequent social media use, and lower resilience and social support,^29^ which corroborates our study.

Social skills are essential to psychosocial well-being in different cultures.^31,32^ However, cultural differences that must be investigated, making it difficult to directly compare studies in different cultures. For example, researchers have argued that people from more individualistic cultures (USA) seem to have more elevated social skills (e.g., due to socialization goals of parents) compared to those in collectivistic cultures,^33^ and these social skills offer benefits to a person’s well-being.

In 2016, social isolation and its implications were studied and their correlation with loneliness was moderate, emphasizing that both were associated with depression, considering genetic factors.^21^ Logistic regression analyses showed that loneliness was more associated with depression when compared with social isolation.^21^ These authors concluded that young adults in social isolation did not necessarily experience loneliness, but those who feel lonely present more signs of depression.^21^ In our study, loneliness during the pandemic was not assessed, neither if the participant lived alone in this period, which could be a limitation of the study and should be further studied. However, the emotional effect during the pandemic was high, especially in females.

## Conclusion

The practice of social isolation during the COVID-19 pandemic in Brazil had social determinants. High levels of psychological and event impacts were detected. The presence of orofacial pain seemed to increase during this health crisis, and individuals form different genders responded differently to the COVID-19 pandemic.
